# Urocortin-1 within the Centrally-Projecting Edinger-Westphal Nucleus Is Critical for Ethanol Preference

**DOI:** 10.1371/journal.pone.0026997

**Published:** 2011-10-28

**Authors:** William J. Giardino, Davelle L. Cocking, Simranjit Kaur, Christopher L. Cunningham, Andrey E. Ryabinin

**Affiliations:** Department of Behavioral Neuroscience and Portland Alcohol Research Center, Oregon Health & Science University, Portland, Oregon, United States of America; Radboud University, The Netherlands

## Abstract

Converging lines of evidence point to the involvement of neurons of the centrally projecting Edinger-Westphal nucleus (EWcp) containing the neuropeptide Urocortin-1 (Ucn1) in excessive ethanol (EtOH) intake and EtOH sensitivity. Here, we expanded these previous findings by using a continuous-access, two-bottle choice drinking paradigm (3%, 6%, and 10% EtOH vs. tap water) to compare EtOH intake and EtOH preference in Ucn1 genetic knockout (KO) and wild-type (WT) mice. Based on previous studies demonstrating that electrolytic lesion of the EWcp attenuated EtOH intake and preference in high-drinking C57BL/6J mice, we also set out to determine whether EWcp lesion would differentially alter EtOH consumption in Ucn1 KO and WT mice. Finally, we implemented well-established place conditioning procedures in KO and WT mice to determine whether Ucn1 and the corticotropin-releasing factor type-2 receptor (CRF-R2) were involved in the rewarding and aversive effects of EtOH (2 g/kg, i.p.). Results from these studies revealed that (1) genetic deletion of Ucn1 dampened EtOH preference only in mice with an intact EWcp, but not in mice that received lesion of the EWcp, (2) lesion of the EWcp dampened EtOH intake in Ucn1 KO and WT mice, but dampened EtOH preference only in WT mice expressing Ucn1, and (3) genetic deletion of Ucn1 or CRF-R2 abolished the conditioned rewarding effects of EtOH, but deletion of Ucn1 had no effect on the conditioned aversive effects of EtOH. The current findings provide strong support for the hypothesis that EWcp-Ucn1 neurons play an important role in EtOH intake, preference, and reward.

## Introduction

Compulsive use of alcohol (ethanol; EtOH) is thought to arise from EtOH-induced adaptations within several neural circuits that together lead to a persistent dysregulation of drug-seeking [1]. Efforts to characterize the maladaptive changes underlying this phenomenon have identified numerous brain regions and neurotransmitter systems that work in concert to drive EtOH reward, excessive EtOH intake, and EtOH withdrawal [2]–[4].

While earlier studies utilized experimenter-administered EtOH to map brain areas thought to be important for sensitivity to the behavioral effects of EtOH, more recent experiments have improved the face validity of this approach (and narrowed the list of candidate brain regions) by implementing self-administration procedures. Specifically, while neural mapping studies showed that experimenter-administered EtOH induced expression of the transcription factor c-Fos in several brain areas [5]-[8], the Edinger-Westphal nucleus (EW) was the only brain area that, across multiple strains and species of rodents, also showed elevated c-Fos expression following oral self-administration of EtOH [9]–[17].

The EW is a compact region within the ventromedial periaqueductal gray that extends along the midline between the caudal division of the ventral tegmental area and the rostral division of the dorsal raphe nucleus. While this nucleus has been historically described as a cholinergic population of preganglionic neurons controlling oculomotor functions, more detailed examinations have revealed that the EW is comprised of two distinct (yet partially overlapping) nuclei, designated EWpg for the preganglionic oculomotor neurons, and EWcp for the centrally-projecting, neuropeptide-containing neurons [18]–[21]. Following the initial studies that characterized the unique sensitivity of the EW to EtOH-drinking, we repeatedly showed that EtOH-induced expression of c-Fos in the EW was restricted almost completely to EWcp neurons containing the neuropeptide Urocortin-1 (Ucn1) [13], [22], [23].

Ucn1 is a member of the corticotropin-releasing factor (CRF) family of endogenous ligands (along with CRF, Ucn2, and Ucn3), and is distinguished from its related peptides by the fact that it binds to both CRF receptor subtypes (CRF-R1, CRF-R2) with high affinity [24], [25]. Given the prior literature implicating a role for CRF systems in excessive EtOH intake following dependence [26] and stress-induced relapse of EtOH-seeking [27], we hypothesized that EWcp-Ucn1 neurons might also contribute toward behavioral phenotypes relevant to alcoholism.

This hypothesis was later validated by data from our laboratory demonstrating that EWcp-Ucn1 protein levels were differentially expressed between rodent lines that had been selectively-bred for divergent EtOH phenotypes. In general, these studies indicated that stronger expression of EWcp-Ucn1 peptide was associated with a genetic predisposition toward high EtOH intake [14], [28], [29] and heightened sensitivity to some (reward, hypothermia, sedation), but not all (locomotor stimulation) EtOH-related phenotypes [22], [30]–[32]. Furthermore, we found that electrolytic lesions of the EWcp in high-drinking C57BL/6J (B6) mice attenuated intake and preference of EtOH (but not sucrose, quinine, saccharin, or saline) in a two-bottle choice (2-BC) drinking paradigm [33], [34]. Together, these studies provided converging lines of evidence to support the claim that EWcp-Ucn1 neurons play an important role in EtOH sensitivity and EtOH consumption [31].

However, because several other neuropeptide systems co-exist with Ucn1 in the EWcp [35]–[39], the possibility remained that EWcp lesion altered EtOH consumption via a Ucn1-independent mechanism (i.e., through a different neuropeptide or receptor expressed in EWcp). Thus, in order to determine whether the effects of EWcp lesion could be attributed specifically to Ucn1, the present studies compared the effects of EWcp lesion on 2-BC intake and preference between mice lacking Ucn1 and their wild-type littermates. In addition, we set out to test whether genetic deletion of Ucn1 would decrease EtOH drinking, EtOH-induced reward, and EtOH-induced aversion in mice containing an intact EWcp. Finally, we also tested whether genetic deletion of CRF-R2 would alter EtOH-induced reward. The findings presented herein shed additional light on the contribution of the EWcp to EtOH intake, and provide further evidence that Ucn1 is an important neuropeptide for mediating the EWcp's effects on EtOH preference and reward.

## Materials and Methods

### Animals

All protocols were approved by the Oregon Health & Science University animal care and use committee (protocol A828), and were performed with strict adherence to the National Institutes of Health Guidelines for the Care and Use of Laboratory Animals. We used single gene mutant mice created from embryonic stem cells that underwent targeted gene inactivation. Ucn1 knockout (KO) mice generated on a 129×1/SvJ x C57BL/6J (B6) background contained a deletion of exon 2 of the *Ucn* gene [40], and CRF-R2 KO mice generated on a 129×1/SvJ x B6 background contained a deletion of exons 3–4 of the *Crhr2* gene [41]. Colonies were maintained by backcrossing onto a B6 genetic background. The Ucn1 KO line was backcrossed onto a B6 background for 10–12 generations, and the CRF-R2 KO line was backcrossed onto a B6 background for 14 generations. KO and wild-type (WT) mice used for these studies were littermates, generated by heterozygous matings. Mice were weaned at 28–32 days of age, isosexually housed, and either underwent surgery at 9–16 weeks of age (EtOH drinking procedures) or underwent behavioral testing at 8–14 weeks of age (EtOH conditioning procedures). Importantly, genetic deletion of either Ucn1 or CRF-R2 does not alter the rate of EtOH elimination [42], [43]. All mice received *ad libitum* access to food (LabDiet 5001; LabDiet, Richmond, IN) and water, with the exception of time spent in the behavioral apparatus (EtOH conditioning experiments only) and remained on a 12 h light-dark schedule (lights on at 0700 h).

Mice on a B6 genetic background are well-known for their high levels of EtOH intake and preference, and are the ideal choice for EtOH drinking studies, particularly 2-BC drinking studies [44]. While mice on a DBA/2J background generally exhibit more robust levels of EtOH place conditioning than mice on a B6 background [45], we chose to use mice on a B6 genetic background in order to produce data that would be comparable to 2-BC drinking experiments, as well as to avoid the time-consuming and expensive process of backcrossing our KO mice onto a DBA/2J background.

### Surgical Procedures

EWcp lesion surgery was performed similar to previous reports [33], [34] in male Ucn1 KO and WT littermate mice. Immediately prior to surgery, mice were given a subcutaneous injection of Rimadyl (Carprofen; 5 mg/kg). Mice were placed under isoflurane anesthesia, secured in a stereotaxic apparatus, and received either electrolytic lesion of the EWcp or sham surgery. For both operations, a small hole was drilled through the skull on the midline (−3.4 mm, A/P; a coordinate that lies halfway along the rostral-caudal axis of the EWcp) and a stainless steel electrode (SNE-300, Rhodes Medical Instruments, Inc., Woodland Hills, CA) was guided down into the EWcp nucleus (−3.9 mm, D/V). The electrode was connected to the positive terminal of a lesion-making device (Model 3500, Ugo Basile, Comerio, Italy). To ground the animal, the negative terminal was attached to the mouse's tail. For sham animals, the electrode remained inactive, but for lesion animals, the electric current (0.4 mA) was activated for five seconds.

Following this procedure, the electrode was removed, the skin was sutured, and animals were single-housed in a cage containing fresh bedding and food, and a single bottle containing tap water. Loss of body temperature was avoided by placing the cage on a heating pad for 30–60 min during the initial recovery period. Following five-nine days of recovery from surgery, mice were given access to two 25 mL glass cylinder bottles (both containing tap water) in order to habituate the animals to drinking from two bottles in the homecage. Importantly, we have previously shown that lesions of EWcp do not produce changes in locomotor activity or the rate of EtOH elimination [33], nor do they produce changes in anxiety-like behavior [34].

### Ethanol Drinking Procedures

Following four days of drinking tap water from two bottles, individually-housed mice underwent a twelve-day EtOH-drinking experiment during which they received 24-hour access to two bottles: one containing tap water, and one containing varying concentrations of EtOH dissolved in tap water. The experiment consisted of three phases during which mice had access to either: 3% EtOH and H2O (Days 1–4), 6% EtOH and H2O (Days 5–8), or 10% EtOH and H2O (Days 9–12). Higher concentrations of EtOH or other palatable fluids were not tested here because earlier studies indicated that EWcp lesion did not affect preference (or avoidance) of several solutions [33], [34]. Mice were weighed and fluid levels from each of the two bottles were recorded on a daily basis between 1000–1200 h. The locations of the bottles on the cages (left vs. right) were alternated daily to avoid the potential confound of an inherent side preference.

### Histology

Immediately following the final day of access to 10% EtOH, mice were euthanized by CO_2_ inhalation. Brains were rapidly dissected, post-fixed overnight in 2% paraformaldehyde in phosphate-buffered saline (PBS), and cryoprotected in 30% sucrose in PBS until saturation. Coronal slices of the midbrain, 30 µm thick, were collected using a CM1850 cryostat (Leica Microsystems) and placed into PBS containing 0.3% NaN_3_ for storage. Six to eight sections spanning the rostral-caudal extent of the EWcp were selected from each animal and underwent Thionin staining. Sections were mounted on clear glass slides, coverslipped, and viewed with the 5× objective on a Leica DM4000 microscope for examination of the location of the lesion (and verification of the absence of damage in sham mice). Images were acquired with the MicroPublisher 3.3 RTV in conjunction with Q-Capture (Q-Imaging, Surrey, BC, Canada). Animals containing lesions that resulted in destruction of a large portion of the EWcp were included in the “Lesion” group, and all sham animals were included in the “Sham” group for statistical analysis of drinking data. An experimenter blinded to the behavioral data was responsible for excluding mice based on incorrect placement of the lesion.

### Statistical Analysis – Ethanol Drinking

Based on the appropriate concentration, EtOH consumption in mL was converted to grams and divided by the animal's body weight to give daily intake scores expressed in grams per kilogram (g/kg). Daily EtOH preference was calculated by dividing EtOH consumption in mL by the total fluid consumption in mL (EtOH consumption + H2O consumption). Total fluid consumption scores were divided by the animal's body weight to give values expressed in mL/kg. Data points across each of the four days of drinking at the 3%, 6%, and 10% concentrations of EtOH were averaged within each animal to produce a single value for EtOH intake (g/kg), EtOH preference, and total fluid consumption (mL/kg) at each of the three phases of the experiment.

Each dependent variable was analyzed by a 2×2×3 repeated measures ANOVA design with genotype (KO, WT) and surgery (Sham, Lesion) as the between-subjects factors, and EtOH concentration (3%, 6%, 10%) as the repeated measure. Significant interactions with EtOH concentration were followed by simple main effect analyses evaluating the impact of surgery and genotype among the three EtOH concentrations. Significant interactions between surgery and genotype were followed by simple main effect analyses evaluating the impact of EtOH concentration and surgery among the two genotypes. Post-hoc comparisons between the four individual groups at each of the three phases of the experiment were made using Bonferroni contrasts corrected for multiple comparisons (significance threshold at *p*<.0083). For all analyses other than post-hoc comparisons, significance threshold was set at *p*<.05. Data are expressed as mean + standard error of the mean (SEM).

### Conditioning Apparatus

The apparatus for EtOH conditioning consisted of four identical boxes measuring 30×15×15 cm that contained six detectors placed 2.2 cm above the floor for acquisition of spatial location and locomotor activity data. The conditioned stimuli consisted of two unbiased tactile cues: “grid” and “hole” floors, which were interchangeable within the apparatus. This allowed the experimenter to arrange the cues in either a “split” configuration (for Pre-Test and Test), or a “matching” configuration (for Conditioning). The apparatus and conditioned stimuli have been described in detail elsewhere [46].

### Ethanol Conditioning Procedures

In the first set of conditioning experiments (EtOH-CPP), male and female Ucn1 and CRF-R2 KO and WT littermate mice (n = 7–15 per line, per sex, per genotype) were tested for the conditioned rewarding effects of EtOH using a slight variant of a well-established, unbiased place conditioning protocol in which pre-session exposure to EtOH results in a significant preference for the EtOH-paired environment [46].

On Day 1 (Pre-Test), mice were weighed and given a saline injection (12.5 mL/kg, i.p.) before being placed into the apparatus containing the two different tactile floor cues (“split” configuration; one floor on each side of the chamber) for 30 min. On Days 2–9, mice underwent daily 5-min conditioning trials. Mice in the “Grid+” subgroup were weighed and injected with EtOH (2 g/kg, 20% v/v, i.p.) immediately before being placed into the apparatus containing the *grid* floor cue (“matching” configuration; same floor on both sides of the chamber). On alternating days, mice were weighed and injected with saline before being placed into the apparatus containing the *hole* floor cue on both sides of the chamber. Mice in the “Grid-“ (or “Hole+”) subgroup were treated in a manner opposite from that of Grid+ mice, such that they were weighed and injected with EtOH prior to being placed into the apparatus containing the *hole* floor cues on both sides, while on alternating days, they were weighed and injected with saline prior to being placed into the apparatus containing the *grid* floor on both sides. On Day 10, all mice were weighed and received a saline injection before being placed into the apparatus containing both floor cues (one on each side) for 30 min.

In the second set of conditioning experiments (EtOH-CPA), male and female Ucn1 KO and WT littermate mice (n = 14–23 per sex, per genotype) were tested for the conditioned aversive effects of EtOH using a slight variant of a well-established protocol in which post-session exposure to EtOH results in a significant aversion of the EtOH-paired environment [46], [47]. The protocol used for EtOH-CPA was identical to that described for EtOH-CPP, except that mice were weighed and injected with either EtOH or saline immediately *after* being removed from the apparatus on Days 1–9. A post-session injection on Day 10 was unnecessary, because the experiment was complete by the end of the behavioral Test session. Importantly, the dose and preparation of EtOH were identical for CPP and CPA experiments.

In order to minimize variation in the conditioning response that could occur based on conditioning subgroup (Grid+ vs. Grid-), conditioning order (EtOH/saline vs. saline/EtOH), and side of the EtOH-paired floor during Pre-Test and Test (left vs. right), all of these variables were fully counterbalanced among all groups in all conditioning experiments.

### Statistical Analysis – Ethanol Conditioning

The percent time spent on the grid floor on Day 10 (Test) relative to Day 1 (Pre-Test) was used as the dependent variable (Δ %Time on Grid Floor). Because three-way ANOVAs of all conditioning experiments yielded no significant main or interacting effects of sex, analyses were collapsed across males and females, and data were analyzed by two-way ANOVA with between-subjects factors of genotype (KO, WT) and subgroup (Grid+, Grid-). Significant interactions between genotype and subgroup were followed by simple main effect analyses evaluating the impact of subgroup separately across the two genotypes. For all conditioning analyses, significance threshold was set at *p*<.05. Data are expressed as mean ± SEM.

## Results

### Histology

**Figure 1:**
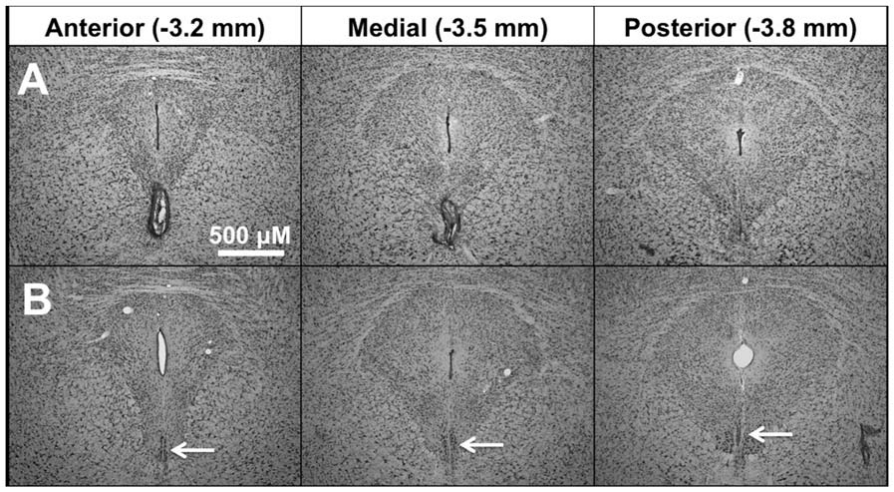
EWcp lesion histology. Representative photomicrographs of Thionin-stained sections from anterior, medial, and posterior EWcp (numbers indicate distance from bregma) taken from mice that underwent (A) successful EWcp lesion surgery or (B) sham surgery. Lesions generally ablated large portions of the anterior and medial EWcp, leaving minimal damage to surrounding tissue. Sham animals displayed no evidence of damage to the EWcp, despite occasional visibility of the electrode tract (posterior panel). White arrows point toward intact EWcp observed in sham animals.

Of the 51 mice that received lesion surgery, Thionin-stained tissue revealed successful targeting and ablation of the EWcp in 29 cases. Importantly, the percentage of successful surgeries was not significantly different between Ucn1 KO and WT mice (16/27 vs. 13/24). Successful lesions were targeted primarily to the anterior and medial EWcp (−3.4 mm from bregma), and caused destruction of this region with minimal damage to surrounding areas (Figure 1A). Animals that received sham surgery showed no evidence of damage to the EWcp or surrounding tissue (Figure 1B).

### EWcp lesion decreases EtOH intake

**Figure 2:**
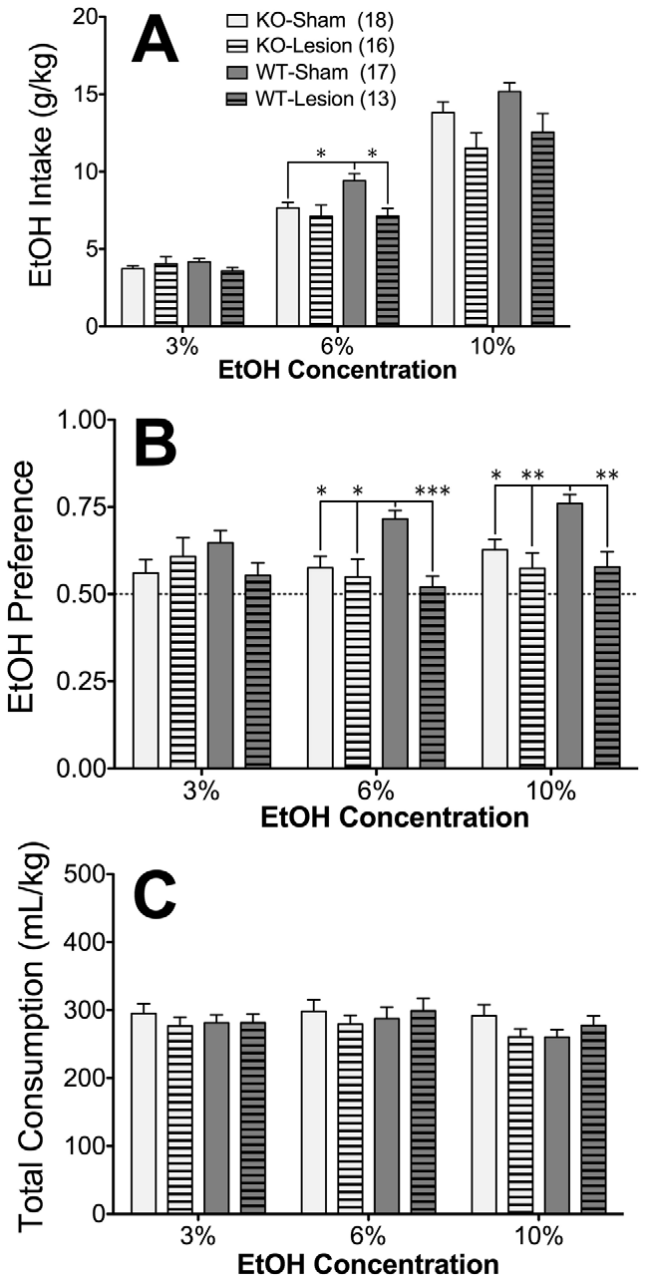
Effects of EWcp lesion on 2-BC EtOH drinking in Ucn1 KO and WT mice. (A) EtOH Intake (g/kg), (B) EtOH Preference, and (C) Total Fluid Consumption (mL/kg) of male Ucn1 KO and WT mice following either sham surgery or EWcp lesion. Asterisks indicate significant difference from the respective WT-Sham group (*p≤.005, **p≤.001, ***p<.0001). Numbers in parentheses indicate group sizes. The same animals contributed to panels A, B, and C.

The initial analysis of EtOH intake indicated differential effects of surgery across the three concentrations (surgery x concentration interaction; *F*_2,120_ = 7.14, *p*<.005; Figure 2A). Follow-up analyses revealed that EWcp lesion significantly reduced intake at the 6% and 10% concentrations of EtOH (simple main effects of surgery; both *F*_1,60_>7.2, both *p*<.01), but not the 3% concentration of EtOH (*p* = .65).

Although EWcp lesion appeared to differentially affect Ucn1 KO and WT mice at the 6% concentration of EtOH, this interaction did not reach significance (*p* = .103). Nevertheless, post-hoc comparisons at the 6% concentration revealed greater intakes in the WT-Sham group relative to both the WT-Lesion group (*p* = .002) and the KO-Sham group (*p* = .005). No other between-group comparisons were significant, indicating that EWcp lesion dampened 6% EtOH intake only in mice expressing Ucn1, and that deletion of Ucn1 dampened 6% EtOH intake only in mice with an intact EWcp.

However, at the 10% concentration of EtOH, the simple main effect of surgery was far from interacting significantly with genotype (*p* = .85), indicating that EWcp lesion was equally effective at reducing intake of 10% EtOH in both Ucn1 KO and WT mice.

### Deletion of Ucn1 decreases EtOH preference only in mice with an intact EWcp, and EWcp lesion decreases EtOH preference only in mice expressing Ucn1

Analysis of EtOH preference indicated that there were differential effects of surgery across the two genotypes (surgery x genotype interaction; *F*_1,60_ = 5.55, *p*<.05). Follow-up analysis revealed that while EtOH preference was significantly dampened by destruction of the EWcp in Ucn1 WT mice (simple main effect of surgery; *F*_1,28_ = 22.28, *p* = .0001), EWcp lesion had no effect on preference in Ucn1 KO mice (*p* = .84). Furthermore, while preference increased in parallel with greater concentrations of EtOH among Ucn1 WT mice (simple main effect of concentration; *F*_2,56_ = 3.24, *p*<.05), Ucn1 KO mice were strikingly resistant to effects of EtOH concentration on preference (*p* = .36).

The conclusion that deletion of Ucn1 dampened EtOH preference only in Sham mice but not Lesion mice, and that EWcp lesion dampened EtOH preference only in Ucn1 WT mice but not Ucn1 KO mice, was supported by post-hoc comparisons at the 6% and 10% concentrations of EtOH, in which the WT-Sham group displayed significantly greater preference than each of the other three groups (all *p*≤.005). No other group comparisons reached significance (all *p*>.25).

### Deletion of Ucn1 and/or lesion of EWcp do not alter total fluid consumption

Total fluid consumption varied significantly across the different concentrations of EtOH (main effect of concentration; *F*_2,120_ = 4.50, *p*<.05; Figure 2C). However, no main or interacting effects with surgery or genotype were found, and no significant post-hoc comparisons were identified between any of the four groups at any of the three EtOH concentrations.

### Deletion of Ucn1 abolishes EtOH-induced CPP

**Figure 3:**
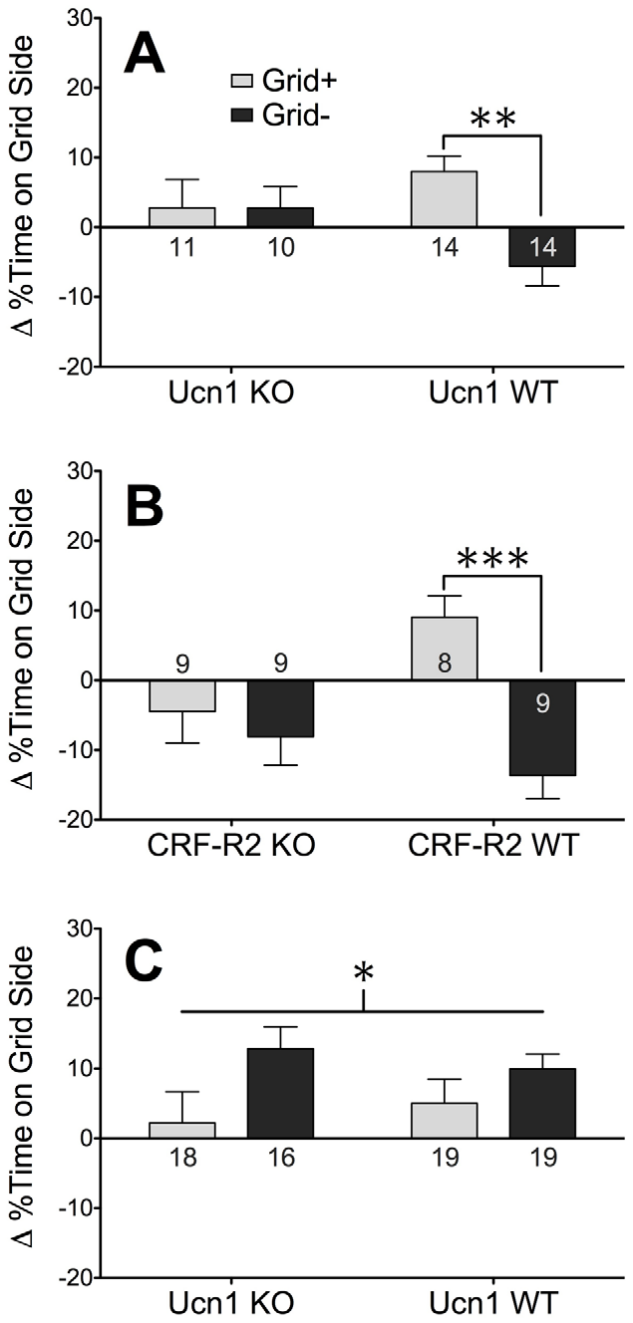
Involvement of Ucn1 and CRF-R2 in EtOH-induced reward and aversion. Graphs show percent change in time spent on Grid Floor between the Pre-Test and the Test following (A) EtOH-CPP in Ucn1 KO and WT mice, (B) EtOH-CPP in CRF-R2 KO and WT mice, and (C) EtOH-CPA in Ucn1 KO and WT mice. Multiple asterisks indicate significant difference between WT subgroups (**p<.001, ***p<.0005). Single asterisk indicates significant main effect of subgroup (*p<.05). Numbers in bars indicate group sizes.

Consistent with previous studies demonstrating the unbiased nature of the tactile floor cues used in our EtOH conditioning studies [48], Ucn1 KO and WT mice spent approximately 50% of their time on the grid floor during the Pre-Test, and this did not differ by genotype or subgroup (data not shown). Following conditioning, preference for the EtOH-paired floor was apparent in Ucn1 WT mice, but not Ucn1 KO mice (genotype x subgroup interaction; *F*_1,45_ = 4.96, *p*<.05; Figure 3A). The conclusion that deletion of Ucn1 abolished EtOH-induced CPP was supported by simple main effect analyses evaluating the impact of subgroup separately across the two genotypes. While strong conditioning was apparent in Ucn1 WT mice (simple main effect of subgroup; *F*_1,26_ = 14.45, *p*<.001), this effect was not apparent in Ucn1 KO mice (*p* = .99).

### Deletion of CRF-R2 abolishes EtOH-induced CPP

Similar to Ucn1 KO and WT mice, CRF-R2 KO and WT mice spent approximately half of their time on the grid floor during the Pre-Test, and this did not differ across genotypes or subgroups (data not shown). Following conditioning, preference for the EtOH-paired floor was apparent in CRF-R2 WT mice, but not CRF-R2 KO mice (genotype x subgroup interaction; *F*_1,31_ = 6.22, *p*<.05; Figure 3B). The conclusion that deletion of CRF-R2 abolished EtOH-induced CPP was supported by simple main effect analyses, in which strong conditioning was apparent in CRF-R2 WT mice (simple main effect of subgroup; *F*_1,15_ = 25.24, *p*<.0005), but not CRF-R2 KO mice (*p* = .56).

### Deletion of Ucn1 does not alter EtOH-induced CPA

In a separate experiment using an EtOH conditioning protocol that produces CPA rather than CPP, Ucn1 KO and WT mice again spent approximately 50% of their time on the grid floor during the Pre-Test, and this did not differ by genotype or subgroup (data not shown). While EtOH conditioning resulted in a significant CPA (main effect of subgroup; *F*_1,68_ = 5.25, *p*<.05; Figure 3C), this effect did not interact significantly with genotype (*p* = .40), indicating that Ucn1 KO and WT mice were equally sensitive to the conditioned aversive effects of EtOH.

## Discussion

The principal findings of the current study were that EtOH intake and preference depended on an interaction between whether or not mice expressed Ucn1, and whether or not mice had received surgical ablation of the EWcp. In addition, we demonstrated that Ucn1 signaling (most likely via CRF-R2) is necessary for the conditioned rewarding effects of EtOH, and that this cannot be attributable to a generalized learning deficit in Ucn1 KO mice. Together, these results indicate that EWcp-Ucn1 neurons influence the magnitude of EtOH intake and preference for EtOH-containing fluids, and that this involvement is likely related to Ucn1's role in mediating sensitivity to the rewarding, but not aversive, effects of EtOH.

Although EWcp lesion and/or deletion of Ucn1 were both capable of attenuating measures of EtOH consumption, it is important to note that these manipulations differentially affected the outcomes of EtOH intake vs. EtOH preference. When examining EtOH intake, our analyses revealed that EWcp lesion reduced drinking in both Ucn1 KO and WT mice. Although examination of the 6% concentration suggested a potential interaction between genotype and surgery, examination of the 10% concentration indicated that EWcp lesion was equally effective at reducing EtOH-drinking in both Ucn1 KO and WT mice. The fact that EWcp lesion decreased 10% intake in mice lacking Ucn1 suggests that other neural systems in the EWcp besides Ucn1 may also contribute to intake of 10% EtOH.

Indeed, the receptor for the orexigenic peptide, ghrelin (growth hormone secretagogue receptor; Ghsr) is densely expressed in the mouse EWcp [36], and our laboratory reported that systemic administration of a Ghsr antagonist not only prevented EtOH-induced neural activity within the EWcp, but also reduced intake of 20% EtOH in a model of binge-like drinking [16]. Furthermore, the receptor for the anorexigenic peptide, leptin (Lepr) is also expressed in the mouse EWcp, and Lepr signaling increases the expression of Ucn1 peptide by directly activating EWcp neurons [39]. In addition, mutant mice that are either leptin-deficient (ob/ob) or leptin-resistant (db/db) showed decreased EtOH preference relative to their wild-type littermates in a 2-BC procedure [49]. These studies suggest that signaling via EW-Ghsr and/or EW-Lepr may be important for EWcp-Ucn1's effects on EtOH preference and reward. Finally, Ucn1 is also highly co-localized in the EWcp with the anorexigenic neuropeptide cocaine- and amphetamine-regulated transcript (CART) [35], [50], and although a role for CART in EtOH-related behaviors has been supported by several studies [51]–[53], the contribution of EWcp-CART neurons to these phenotypes has not yet been thoroughly examined.

The EWcp also expresses high levels of the peptides cholecystokinin, nesfatin-1, and neuropeptide B [54]–[57] . Since these peptides have anorexic properties, it is logical to assume that they could also contribute to EWcp's involvement in consummatory behaviors. This would be in agreement with our observations that EWcp lesions can alter fluid consumption [33], [34]. However, the reductions in EtOH intake observed here were not simply due to a non-specific decrease in consumption, because the total volume of fluid consumption was not affected (Fig. 2C), and the effect on EtOH preference was dependent on both Ucn1 genotype and the type of surgery, as discussed below.

In contrast to effects on EtOH intake, analysis of EtOH preference revealed a significant interaction between surgery and genotype. Post-hoc comparisons at concentrations of both 6% and 10% confirmed that deletion of Ucn1 reduced preference only in mice with an intact EWcp, and that lesion of EWcp reduced preference only in mice expressing Ucn1. These findings provide strong evidence that EWcp-Ucn1 neurons are necessary for driving high EtOH preference, and suggest that our previous report of dampened EtOH preference in EWcp-lesioned B6 mice can be attributed primarily to the reduction of Ucn1-positive terminals within EWcp target regions [33]. The potential dissociation between Ucn1's involvement in regulation of EtOH *preference* and the contribution of other EWcp peptide systems to regulation of EtOH *intake* is intriguing, and requires further investigation.

One potential caveat of examining genetically-engineered KO mice is that observed effects can sometimes be better attributed to developmental compensations within systems related to the deleted gene, rather than to the absence of the gene itself. In fact, one possible explanation for why we uncovered a role for Ucn1 in EtOH *preference*, but not EtOH *intake* is that this effect was masked by developmental compensations in Ucn1 KO mice. However, because Ucn1 is the only component of the CRF system that is expressed in the EWcp, and because the EWcp is the primary site of Ucn1 expression in the mammalian brain [58]-[60], our observation that EWcp lesion differentially affected EtOH preference in Ucn1 KO and WT mice suggests that the effects of Ucn1 deletion on EtOH-related behaviors can be primarily ascribed to the actions of EWcp-Ucn1 neurons. Furthermore, we have previously shown that Ucn1 is only expressed postnatally in the EWcp [61], limiting the potential impact of compensations on development. Indeed, converging lines of evidence provide additional support for the involvement of EWcp-Ucn1 neurons in EtOH sensitivity [14], [31].

The current data complement a wealth of existing literature on the contribution of specific components of the CRF system to EtOH-related behaviors. Importantly, the function of EWcp-Ucn1 neurons appears to differ substantially from the role of CRF-containing neurons in the central nucleus of the amygdala (CeA). While we speculate that the involvement of EWcp-Ucn1 neurons in EtOH preference and reward predominates during the initial stages of the addiction process, CeA-CRF neurons are thought to be integral for the transition to EtOH addiction and the negative reinforcement processes that prevail during dependence and withdrawal [62], [63].

For example, excessive release of CeA-CRF occurs during EtOH withdrawal [64], [65], CeA-CRF mRNA is upregulated following EtOH dependence [66], [67], and CRF's ability to release GABA from CeA interneurons is potentiated in EtOH-dependent rats [67]. Although we have not yet ruled out a potential role for Ucn1 in EtOH dependence, the current results support a general framework in which CRF and CRF-related peptides display unique relationships with distinct aspects of the addiction process. Indeed, while CRF is required for EtOH-induced psychomotor sensitization and binge-like EtOH intake, it appears that Ucn1 is not critical for these behaviors [42], [43].

It is important to note that although Ucn1 binds with high affinity to both CRF receptors [24], [25], it remains unclear specifically which EtOH-related behaviors involve EWcp-Ucn1 actions at CRF-R1 vs. CRF-R2. Numerous reports have demonstrated that genetic deletion or pharmacological blockade of CRF-R1 decreases EtOH consumption [42], [68]–[70], and these effects are generally more pronounced in animals with an extensive history of EtOH exposure [67], [71]–[77].

In contrast, several studies have concluded that CRF-R2 signaling acts to *decrease* EtOH consumption [70], [78], [79]. However, CRF-R2 regulation of behavior is often reported as bi-directional [80]–[82], and one study demonstrated that intra-CeA CRF-R2 activation had opposing effects on EtOH self-administration in dependent vs. non-dependent rats [83]. Indeed, the observations that deletion of CRF-R2 blocked EtOH-CPP (Fig. 3B) and protected against prolonged increases in EtOH preference following stress [84] indicate that the precise role of CRF-R2 signaling in EtOH-related behavior may rely on a complex interaction between several experimental variables.

Because earlier studies from our laboratory found that EWcp lesion decreased the number of Ucn1-positive terminals in the lateral septum and dorsal raphe nucleus [33], and because CRF-R2 expression is enriched in those areas relative to CRF-R1 [85], [86], we have hypothesized that EWcp-Ucn1 mediates its effects on EtOH-related behaviors primarily via CRF-R2 rather than CRF-R1. Although this idea is supported by our observation that Ucn1 KO mice and CRF-R2 KO mice are both resistant to EtOH-CPP (suggesting that Ucn1 acts via CRF-R2 to mediate the conditioned rewarding effects of EtOH), this hypothesis awaits further confirmation. Nevertheless, accumulating evidence suggests that the importance of urocortin peptides and CRF-R2 signaling in mediating emotional states [87], [88] and drug-induced behaviors [89], [90] remains underappreciated.

In summary, we utilized two complementary methods (genetic knockout of Ucn1 and electrolytic lesion of EWcp) in an examination of 2-BC EtOH consumption, and accompanied these studies with place conditioning experiments capable of dissociating sensitivity to the rewarding vs. aversive effects of EtOH. Taken together, our results implicate the EWcp in EtOH intake, EWcp-Ucn1 neurons in EtOH preference, and Ucn1/CRF-R2 in EtOH-induced reward. Future studies examining different drinking paradigms, different concentrations of EtOH, potential effects of dependence, and additional EtOH-related behaviors will assist in delineating the specific components of the CRF system (and the specific neural substrates) that work in concert to drive the progression of EtOH addiction.
